# Evaluation of lipid matrix microencapsulation for intestinal delivery of thymol in weaned pigs

**DOI:** 10.1093/tas/txz176

**Published:** 2019-11-22

**Authors:** Janghan Choi, Lucy Wang, Emily Ammeter, Ludovic Lahaye, Song Liu, Martin Nyachoti, Chengbo Yang

**Affiliations:** 1 Department of Animal Science, University of Manitoba, Winnipeg, Manitoba, Canada; 2 Department of Biosystems Engineering, University of Manitoba, Winnipeg, Manitoba, Canada; 3 Jefo Nutrition Inc., Saint-Hyacinthe, Quebec, Canada

**Keywords:** essential oils, in vivo release, in vitro release, microencapsulation, pelleting, pigs

## Abstract

Essential oils (EO) are defined as plant-derived natural bioactive compounds, which can have positive effects on animal growth and health due to their antimicrobial and antioxidative properties. However, EO are volatile, can evaporate quickly, and be rapidly absorbed in the upper gastrointestinal tract. Also, due to their labile nature, the stability of EO during feed processing is often questionable, leading to variations in the final concentration in feed. Encapsulation has become one of the most popular methods of stabilizing EO during feed processing, storage, and delivery into the lower gut. The objectives of the present study were to 1) evaluate the stability of thymol microencapsulated in combination with organic acids in commercially available lipid matrix microparticles during the feed pelleting process and storage; 2) validate and demonstrate the slow release of thymol from the lipid matrix microparticles in a simulated pig gastric fluid (SGF) and a simulated pig intestinal fluid (SIF); and 3) evaluate in vivo release of thymol from the lipid matrix microparticles along the pig gut. The results showed that thymol concentration was not significantly different in the mash and pelleted feeds (*P* > 0.05). In the in vitro study, 26.04% thymol was released in SGF, and the rest of the thymol was progressively released in SIF until completion, which was achieved by 24 h. The in vivo study showed that 15.5% of thymol was released in the stomach, and 41.85% of thymol was delivered in the mid-jejunum section. Only 2.21% of thymol was recovered in feces. In conclusion, the lipid matrix microparticles were able to maintain the stability of thymol during a feed pelleting process and storage and allow a slow and progressive intestinal release of thymol in weaned pigs.

## INTRODUCTION

During the weaning phase, piglets frequently have diarrheic symptoms and other intestinal disturbances, which can result in decreased growth performance and mortality (Yang et al., 2015; Hassan et al., 2018). Traditionally, antibiotic growth promoters (AGP) were used to reduce the complications associated with weaning. However, there is a concern regarding the transmission and the proliferation of resistant bacteria via the food chain, which has induced regulations and restrictions of the use of AGP in animal feed in many countries (Zeng et al., 2015b). Various AGP alternatives have been developed and practically used in the swine industry (Cheng et al., 2014).

Essentials oils (EO), natural bioactive compounds obtained from plants, are known to have antibacterial, antiviral, antifungal, and antioxidative properties and have traditionally been used as complementary or alternative medicines to improve human health or cure human diseases (Kim et al., 2008; Brenes and Roura, 2010). With the identification of active components in plant extracts and some progress in the mechanistic studies of these components in animals, there has been an increase of studies in pursuit of using EO to substitute AGP in animal diets (Li et al., 2012). Many studies found that various EO (e.g., thymol, cinnamaldehyde, and eugenol) could improve growth performance (Manzanilla et al., 2006; Nofrarias et al., 2006), gut immune system (Su et al., 2018), gut morphology (Xu et al., 2018), and gut microbiota (Zeng et al., 2015a). However, the lipophilic and volatile properties of EO are obstacles that must be considered when including EO in pig feed (Omonijo et al., 2018b). Due to their volatile properties, EO may be absorbed into feed components or air-dried and evaporated during feed processing (e.g., pelleting), leading to reduced potency (Si et al., 2006). Several studies indicated that EO were mainly or almost entirely absorbed in the stomach and the proximal small intestine of piglets after oral intake (Michiels et al., 2008). Thus, the majority of the EO, without proper protection, will be lost during feed processing and delivery to the pig gut and may not be able to reach the lower gut of pigs where most pathogens reside and propagate (Chen et al., 2017), which will reduce the profitability of feed mills and become one of the major barriers for EO application in feed. Thus, it is crucial to develop an effective and practically feasible delivery method for the use of EO in the feed.

Encapsulation, which provides a physical barrier for bioactive compounds and separates the core material from the environment until release, is thought to improve the stability of bioactive compounds and enable the slow release of EO in animals (Vidhyalakshmi et al., 2009). Lipid matrix microencapsulation has been popularly used to deliver bioactive compounds (e.g., EO, organic acids, and vitamins) to the animal’s gut (Liu et al., 2017a; Gottschalk et al., 2018; Yang et al., 2018; Kaur et al., 2019; Yang et al., 2019). However, there is a lack of information on the stability of EO during feed processing and storage and the intestinal release of EO from the lipid matrix microparticles in animals. This study hypothesized that EO embedded (microencapsulated) in a commercially available lipid matrix microparticles as a blend of EO and organic acids (OA) will maintain their stability during the pelleting process and storage and EO may be slowly released in the pigs’ gut. Therefore, the objectives of this research were to evaluate the stability of thymol in the lipid matrix microparticles during feed pelleting and feed storage and to determine the intestinal release of thymol using in vitro and in vivo approaches.

## MATERIALS AND METHODS

### Materials

Thymol (≥98.5%), α-methyl-trans-cinnamaldehyde (≥98%), hexane (high performance liquid chromatography grade, 95%), and pepsin derived from porcine gastric mucosa (≥250 units/mg), bile salts, pancreatin originated from porcine pancreas (≥3 United States Pharmacopeia), and titanium dioxide (≥99% trace metal basis) were purchased from Sigma-Aldrich (Oakville, Ontario, Canada). A blend of EO and OA was embedded in lipid matrix microparticles (Jefo Nutrition Inc., Saint-Hyacinthe, Quebec, Canada). The components of the lipid matrix microparticles were hydrogenated vegetable oil for the matrix material and fumaric acid, sorbic acid, malic acid, citric acid, soya lecithin, thymol, vanillin, and eugenol as active ingredients embedded (microencapsulated) within the lipid matrix.

### Thymol Stability in the Lipid Matrix Microparticles During Feed Pelleting Process and Storage

The stability of thymol in the lipid matrix microparticles was determined during the pelleting process. A wheat-soybean meal (SBM) basal diet was formulated as shown in Table 1. The treatments included: 1) a control mash basal diet control mash feed (CM), 2) CM + 0.2% of the lipid matrix microparticles Essential oil mash feed (EOM), 3) CM pelleted control pelleted feed (CP), and 4) EOM pelleted Essential oil pelleted feed (EOP). The lipid matrix microparticles were premixed with corn (approximately 8 kg) before being added to the whole diets. The pelleting process was conducted with a Master Model California Pellet Mill (California Pellet Mill Co., San Francisco, CA, USA) at the Glenlea Swine Research Unit at the University of Manitoba. The air temperature during conditioning and pelleting was measured with a noncontact infrared thermometer (Fluke 62 mini infrared thermometer, Fluke Corporation, Everett, WA, USA). Conditioning before pelleting was conducted at 69–74 °C by directly adding steam to a mixer where feed and steam were thoroughly mixed and, after 4 s, the first feed particles moved to the pelleting part. The steam and feed mixture were pressed with a pressor that has a 4-mm diameter and 10-mm length and the pelleting temperature reached to 61 °C. The total pelleting time of each batch was less than 2 min to pellet 50 kg of feed. Six samples were obtained from mash feed, and 6 samples after pelleting were collected. Every batch, the mash feed mixing was followed by the pelleting procedure. The pelleting process was conducted independently three times. The samples were kept at −80°C until further analyses.

**Table 1.** The composition of a wheat-SBM basal diet for the feed pelleting experiment (kg, as-fed basis)

Ingredients	kg
Wheat	400
Barley	60
Corn	250
Soybean meal (48% crude protein)	215
Soybean oil	10
Fish meal	40
Limestone	10
Vitamin–minerals premix^1^	14
L-lysine HCl	1
Total	1,000
Calculated net energy and nutrient content (g/kg)	
Net energy (kcal/kg)	2,272
Crude protein, %	22.0

^1^Supplied the following per kilogram of diet: 2,200 IU vitamin A, 220 IU D3, 16 IU E, 0.5 mg vitamin K, 1.5 mg vitamin B1, 4 mg vitamin B2, 12 mg calcium pantothenate, 600 mg choline chloride, 30 mg niacin, 7 mg pyridoxine, 0.02 mg vitamin B12, 0.2 mg biotin, 0.3 mg folic acid, 0.14 mg calcium iodate, 6 mg copper sulphate, 100 mg ferrous sulfate, 4 mg manganese oxide, 0.3 mg sodium selenite, and 100 mg zinc oxide.

The stability of thymol in the lipid matrix microparticles during feed storage was measured for 12 wk at room temperature. The feeds from the third batch of EOM and EOP were used in the experiment. Six samples (400 g of feed) were taken from the EOM and EOP. Each feed sample was placed in an opened zip bag, and a total of 12 bags were stored in two plastic containers (45 cm × 30 cm × 40 cm) with the closed lid. The plastic containers were stored at a temperature of 23–24 °C and a relative humidity of 25%–30%. At the time points of 0, 1, 3, 6, 9, and 12 wk, 25 g of feed were obtained from each bag and then stored at −80°C to minimize thymol evaporation until further analyses.

### In Vitro Release of Thymol in Simulated Gastric and Intestinal Fluids

**Figure 1.**
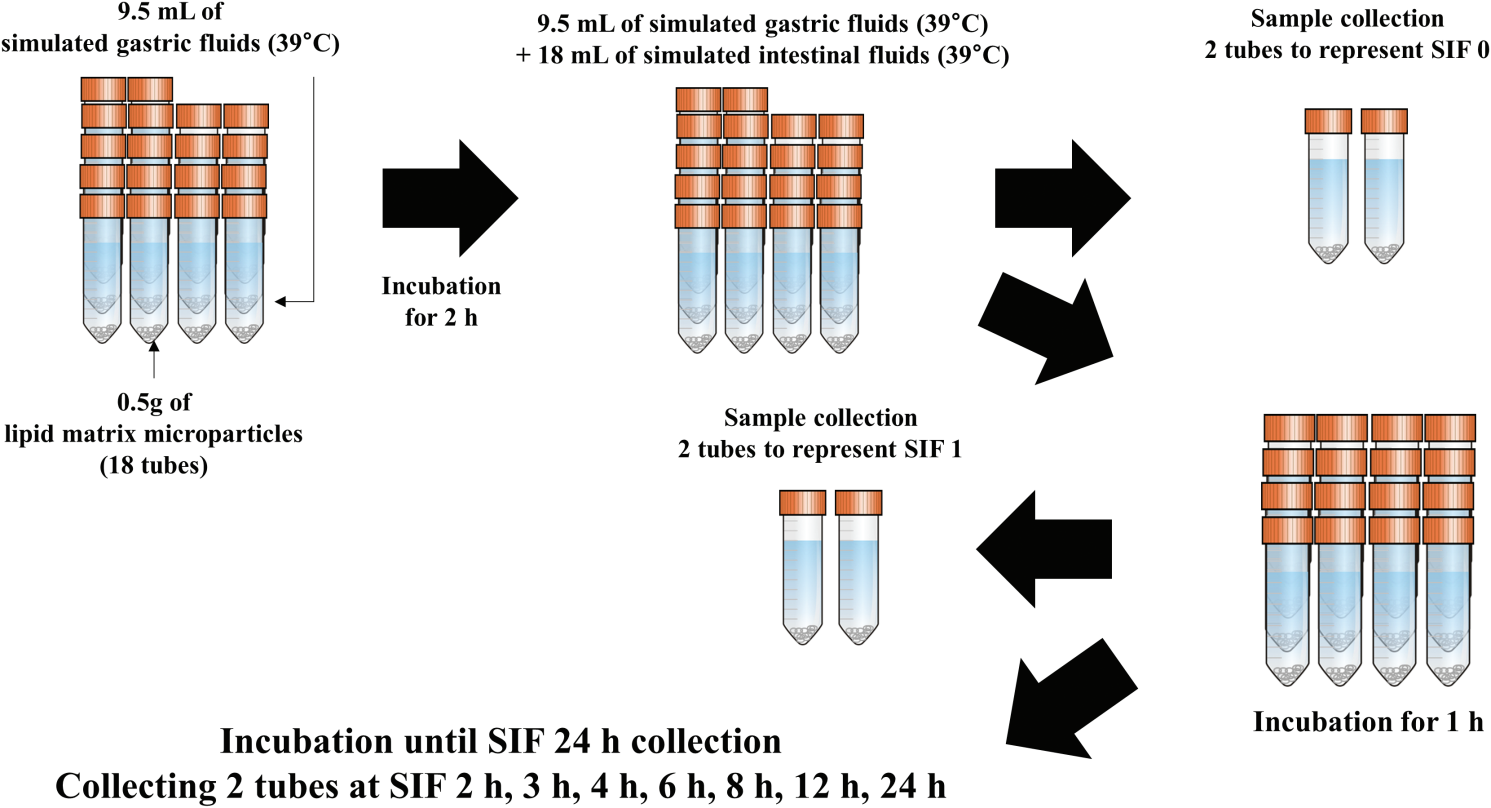
The flow diagram of the in vitro release profile study. The mixture of 9.5 mL of prewarmed simulated gastric fluid (39 °C) and 0.5 g of the lipid matrix microparticles was added into each tube (total 18 tubes) and then incubated for 2 h at 39 °C. After that, 18 mL of prewarmed simulated intestinal fluid (39 °C) was added into the tubes. Two tubes were collected to represent SIF 0. After 1 h incubation, two tubes were collected to represent SIF 1. The rest of the tubes were incubated until SIF 24 h collection and two tubes were collected to represent each time point.

The *in vitro* release profile of thymol in the lipid matrix microparticles was determined using a simulated pig gastric fluid (SGF) and a simulated pig intestinal fluid (SIF). Both SGF and SIF were prepared according to the methods described by Minekus et al. (2014) with some modifications. The SGF contained 47.2 mmol/L NaCl, 25 mmol/L NaHCO_3_, 6.9 mmol/L KCl, 0.9 mmol/L KH_2_PO_4_, 0.5 mmol/L (NH_4_)_2_CO_3_, 0.1 mmol/L MgCl_2_(H_2_O)_6_, 0.15 mmol/L CaCl_2_(H_2_O)_2_, and 2000 U/mL pepsin originated from porcine gastric mucosa. The SIF contained 85 mmol/L NaHCO_3_, 38.4 mmol/L NaCl, 6.8 mmol/L KCl, 0.8 mmol/L KH_2_PO_4_, 0.33 mmol/L MgCl_2_(H_2_O)_6_, 0.6 mmol/L CaCl_2_(H_2_O)_2_, 10 mM bile salts, and 1% (by vol.) pancreatin originated from porcine pancreas (Liu et al., 2017b). The pH of SGF and SIF was adjusted to 3.0 and 7.0, respectively, using HCl or NaOH. The mixture of 9.5 mL of prewarmed SGF (39 °C) and 0.5 g of the lipid matrix microparticles was added into each tube and then incubated at 200 rpm for 2 h at 39 °C. After that, 18 mL of prewarmed SIF (39 °C) was added into the tubes and pH was adjusted to 7.0. Then the tubes were horizontally incubated at 200 rpm for 24 h at 39 °C using a forced-convection laboratory oven (Heratherm, Thermo Scientific Inc., Waltham, MA, USA) and a shaker (MaxQ 2508, Thermo Scientific Inc.). At SIF 0 (SGF 2 h + SIF 0), 1 (SGF 2 h + SIF 1 h), 2, 3, 4, 6, 8, 12, and 24 h, two samples (i.e. two tubes) were taken out to represent each time point and the pH of each sample was adjusted to 5.0 to minimize enzyme activities and the samples were then stored at –20 °C until further analyses (Figure 1). The two tubes collected for each time point were considered as the technical replicates and the in vitro release profile experiment was conducted in triplicates.

### In Vivo Recovery Rate Along the Gut of Weaned Pigs

The experimental and animal care protocol (F17-018, AC11280) was reviewed and approved by the Animal Care Committee of the University of Manitoba and the pigs were cared for in accordance with the Canadian Council on Animal Care (CCAC) guidelines (CCAC, 2009). A total of 12 male piglets (TN Tempo × TN70; 9.86 ± 0.52 kg; 28 d) were obtained from the Glenlea Swine Research Unit at the University of Manitoba and housed individually after 4 d of group adaptation period (days 1–5) in a temperature-controlled room in the T.K. Cheung Centre for Animal Science Research at the University of Manitoba. Room temperature was maintained at 29 ± 1 °C during the first week and then reduced by 1.5 °C for the rest of the experimental period. Piglets were randomly allotted to two treatments in a completely randomized design (*n* = 6): 1) a control corn-SBM basal diet (CF) and 2) a corn-SBM basal diet supplemented with 6 g/kg lipid matrix microparticles Essential oil feed (EOF). The corn-SBM basal diets were formulated to meet or exceed National Research Council (2012) nutrient specifications for pigs weighing 6–10 kg body weight (BW; Table 2). Zinc oxide was added in the diets to prevent diarrhea in pigs and titanium dioxide (3 g/kg) was added as an inert marker to calculate the thymol recovery rate in the different gastrointestinal sections. All pigs had free access to water and feed during the whole experimental period and all experimental diets were provided in a mash form. On days 8 and 9, feces were collected. On day 9, final BW and feed intake were measured and, thereafter, the pigs were anesthetized by an intramuscular injection of ketamine:xylazine (20:2 mg/kg BW) and euthanized by intravenous injection of sodium pentobarbital (110 mg/kg BW). The whole organs of the gastrointestinal tract were removed from the carcass and the digesta samples from the stomach, mid-jejunum (350–450 cm from the stomach-duodenum junction), ileum (upper 0–80 cm of the ileum-cecum junction), cecum, and colon (lower 20 cm from the ileum-cecum junction) were collected into different sterilized containers (Adeola and King, 2006). The samples of collected digesta were kept at −20 °C to be freeze-dried later. The individual pig was considered as the experimental unit.

**Table 2.** The composition of diets used for the in vivo release experiment (kg, as-fed basis)

Ingredients	Control diet	Encapsulated essential oils diet
Corn	477.62	471.62
Soybean meal (48% crude protein)	160.00	160.00
Whey permeate	124.22	124.22
X-SOY600^1^ (60% crude protein)	110.00	110.00
Fish meal	65.73	65.73
Soybean oil	15.00	15.00
Calcium (limestone)	14.32	14.32
Biofos 21%^2^	5.73	5.73
Salt—bulk fine	5.00	5.00
Zinc oxide 72%	3.19	3.19
Vitamin-mineral premix^3^ (1%)	10.00	10.00
L-lysine HCl 78%	2.83	2.83
DL-methionine 99%	1.52	1.52
Threonine	1.32	1.32
L-tryptophan	0.51	0.51
Titanium dioxide (TiO_2_)^4^	3.00	3.00
Lipid matrix microparticles^5,6^	0.00	6.00
Total	1,000.00	1,000.00
Calculated net energy and nutrient content (g/kg)		
Net energy (kcal/kg)	2,475	2,459
Crude protein (%)	22.4	22.3

^1^Soy protein concentration (CJ Selecta, Goiania, Goiás, Brazil).

^2^Ca, 21%; P, 17% (The Mosaic Co., Plymouth, MN).

^3^Supplied the following per kilogram of diet: 2,200 IU vitamin A, 220 IU D3, 16 IU E, 0.5 mg vitamin K, 1.5 mg vitamin B1, 4 mg vitamin B2, 12 mg calcium pantothenate, 600 mg choline chloride, 30 mg niacin, 7 mg Pyridoxine, 0.02 mg vitamin B12, 0.2 mg biotin, 0.3 mg folic acid, 0.14 mg calcium iodate, 6 mg copper sulphate, 100 mg ferrous sulfate, 4 mg manganese oxide, 0.3 mg sodium selenite, and 100 mg zinc oxide.

^4^Titanium dioxide (TiO2; Sigma-Aldrich, Oakville, Ontario, Canada).

^5^Lipid matrix microparticles including hydrogenated vegetable oil, fumaric acid, sorbic acid, malic acid, citric acid, soya lecithin, thymol, vanillin, and eugenol (Jefo, Saint-Hyacinthe, Quebec, Canada).

^6^The lipid matrix microparticles were premixed in corn (approximately 8 kg) before being added to the whole diet.

### Gas Chromatographic Determination of Thymol

Thymol extraction from the feed or digesta samples was conducted according to the methods described by Folch et al. (1957) and Ndou et al. (2018) with some modifications. Samples were freeze-dried and finely ground with a coffee grinder (Applica Consumer Products Inc., Miami Lakes, FL, USA) and 1 g of sample was weighed and added to a 50-mL glass tube. Twenty milliliters of a mixture of chloroform/methanol (2:1, by vol.) and internal standard (α-methyl-trans-cinnamaldehyde) were added and shaken for 1 h to break down the lipid matrix microparticles and absorb thymol in the mixture. After shaking, 5 mL (25% by vol.) of water was added to separate the chloroform and methanol phase and the sample was centrifuged at 750 × *g* for 15 min at 4 °C. The chloroform phase was obtained with a Pasteur pipette (Fisher Scientific, Hampton, NH, USA) and was filtered with a filter paper (P5, Fisher Scientific) and dried under nitrogen gas (N_2_) flux using an N-EVAP 112 evaporator (Organomation Associates Inc., Berlin, MA, USA) at 37 °C. Methylation was done according to the method described by Ichihara and Fukubayashi (2010). Toluene (0.2 mL), methanol (1.5 mL), and 8% HCl (0.3 mL) were added sequentially and the mixture was vortexed and incubated at 45 °C overnight. After the overnight incubation, the solution was evaporated under N_2_ flux using an N-EVAP 112 evaporator (Organomation Associates Inc.). Hexane (2 mL) was added to dissolve thymol and water (2 mL) was added to wash hexane and then the tubes were vortexed and centrifuged at 750 × *g* for 15 min at 4 °C. Finally, the 2 mL of the hexane phase was obtained, and thymol content was analyzed by gas chromatography-flame ionization detector (GC-FID).

Samples from the in vitro release experiment were thawed at room temperature and centrifuged at 4,700 × *g* for 20 min at 4 °C and the supernatant was filtered with a filter paper (P5, Fisher Scientific) and the filtered quantity of the supernatant was recorded. The filtered supernatant was mixed with 15 mL of hexane with internal standard using a rotator (Rotator AG, FINEPCR, Gunpo, Gyeonggi-do, Korea) for 1 h and centrifuged at 750 × *g* for 10 min at 4 °C and the hexane phase was obtained by a Pasteur pipette (Fisher Scientific). The obtained hexane was methylated as described above (Ichihara and Fukubayashi, 2010) and the samples were analyzed by GC-FID.

The amount of thymol was determined by GC-FID. The samples were separated on a CP Select Fames column (100-m × 0.25-mm diameter and 0.25-μm film thickness; Varian Canada, Mississauga, Ontario, Canada) using a Bruker 450 GC with FID (Varian Canada). The temperature program was 70 °C for 2 min, the temperature was raised to 175 °C at 25 °C/min, held for 20 min, raised to 215 °C at 1.5 °C/min, held for 10 min, and raised to 250 °C at 20 °C/min and held for 3 min and the total run time was 67.62 min. Samples were run with a 20:1 split ratio and 0.8 mL/min column flow. The temperature detector was 290 °C and hydrogen was used as the carrier gas.

### Calculation of Thymol Concentrations and Recovery Rates

The thymol concentration was calculated based on the peak area ratio between thymol (specific compound of interest) and α-methyl-trans-cinnamaldehyde (internal standard) as follows (FID, 2003):

$$\begin{array}{l} Thymol\;concentration\;(mg/kg)=\;\frac{AMOUNT_{IS}\times AREA_{SC}\times IRF_{SC}}{AREA_{IS\;}} \end{array}$$

where IS = internal standard, SC = specific compound of interest, and IRF = internal response ratio between IS and SC.

According to Zhang et al. (2016), the recovery rate of thymol in the different gastrointestinal segments was calculated by analyzing thymol and titanium dioxide contents in feed or digesta. Samples for titanium analysis were prepared according to the method proposed by Lomer et al. (2000) and the titanium concentration was determined using an inductively coupled plasma spectrometer (Vista-MPX; Varian Canada). Thymol recovery rate was calculated based on the following equation (Zhang et al., 2016):

$$\begin{array}{l} RECOVERY_{THYMOL}(\%)=\;\left[\frac{\left(MARKER_{DIET}\times THYMOL_{DIGESTA}\right)}{\left(THYMOL_{DIET}\times MARKER_{DIGESTA}\right)}\right]\times 100 \end{array}$$

### Statistical Analyses

GraphPad Prism 7 (GraphPad Software, Inc., San Diego, CA, USA) was used to perform statistical analyses. In the pelleting experiment, the differences in thymol content between the EOM and EOP were analyzed by an unpaired *t-*test. In the stability experiments, total thymol contents were compared by one-way analysis of variance (ANOVA) followed by a Tukey’s test. For the in vitro release experiment, a curve fitting program (Padé approximant) was used. In the in vivo release experiment, the mean and SEM were calculated. Data in all figures are shown as means ± SEM. For all statistical analyses, *P* < 0.05 was considered significant.

## RESULTS

**Figure 2.**
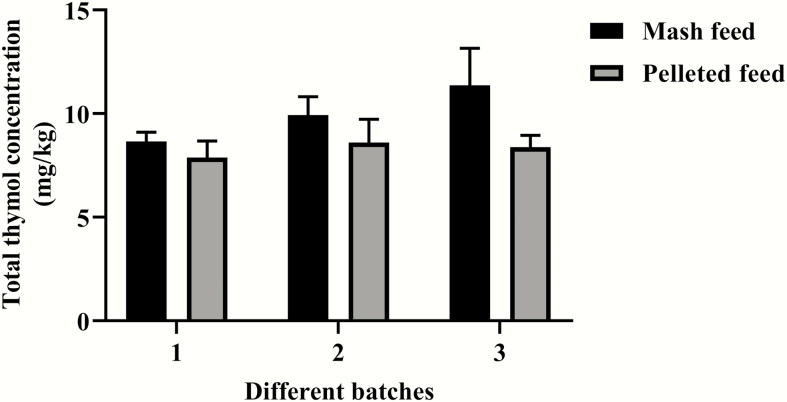
Effect of feed pelleting process on total thymol content in a diet supplemented with or without thymol microencapsulated in the lipid matrix microparticles. Total thymol content in the diets was analyzed by GC-FID. Thymol was not detectable in the diets not supplemented with thymol microencapsulated in the lipid matrix microparticles (both mash and pelleted feeds). Each value represents the mean ± SEM, *n* = 6. Three independent batches were conducted.

**Figure 3.**
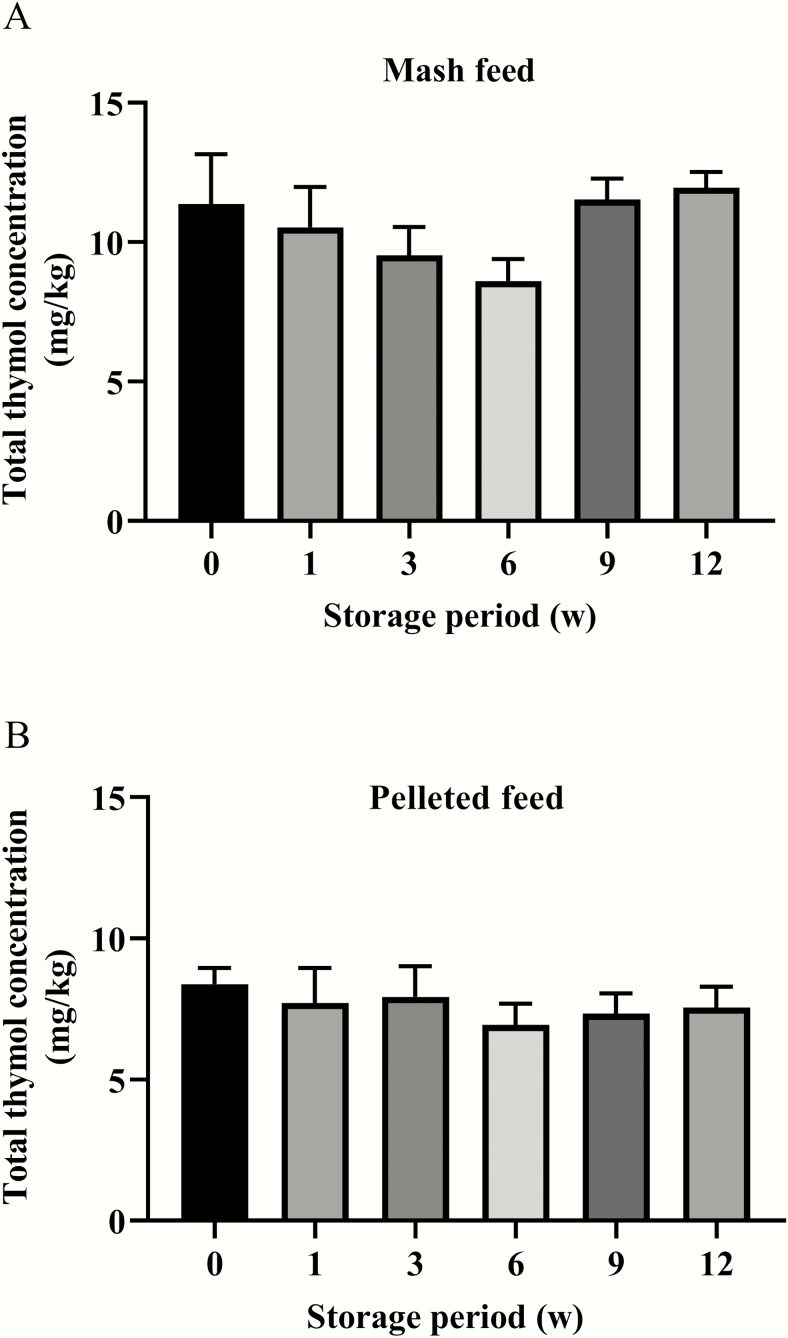
The stability of thymol microencapsulated in the lipid matrix microparticles in the mash feed (A) and pelleted feed (B) during storage. Mash and pelleted feeds supplemented with thymol microencapsulated in the lipid matrix microparticles were stored at room temperature (22–24 °C) and 20%–30% humidity for 12 wk. Each value represents the mean ± SEM, *n* = 6.

The wheat-SBM basal diets either not supplemented or supplemented with thymol microencapsulated in the lipid matrix microparticles were pelleted at up to 74 °C. Thymol was not detectable in the nonsupplemented diets (mash feed and pelleted feed). As shown in Figure 2, there was no difference in the thymol content between EOM and EOP in the three different batches (*P* > 0.05) (Figure 2). As shown in Figure 3, the total amount of thymol in both EOM and EOP did not change during the studied periods (12 wk; *P* > 0.05).

**Figure 4.**
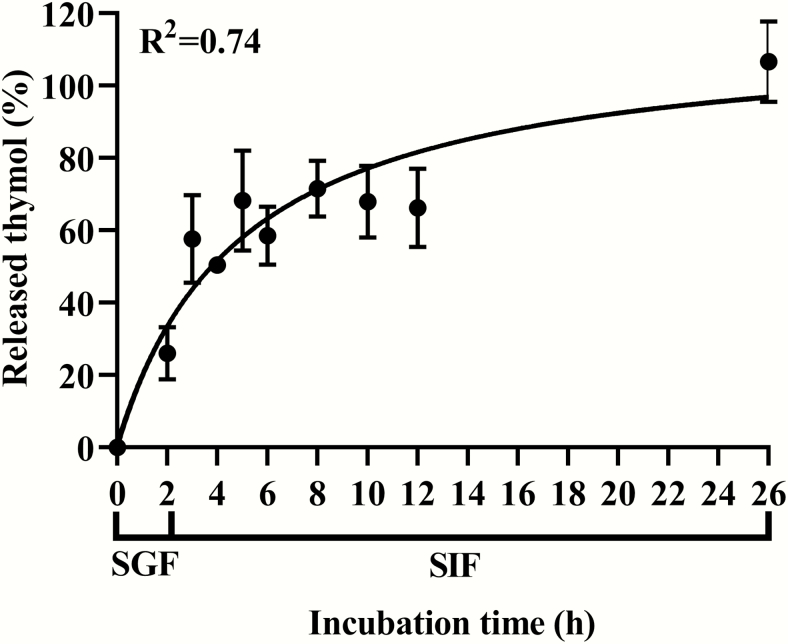
In vitro release profile of thymol from the lipid matrix microparticles in SGF and SIF. Each value represents the mean ± SEM, *n* = 3.

**Figure 5.**
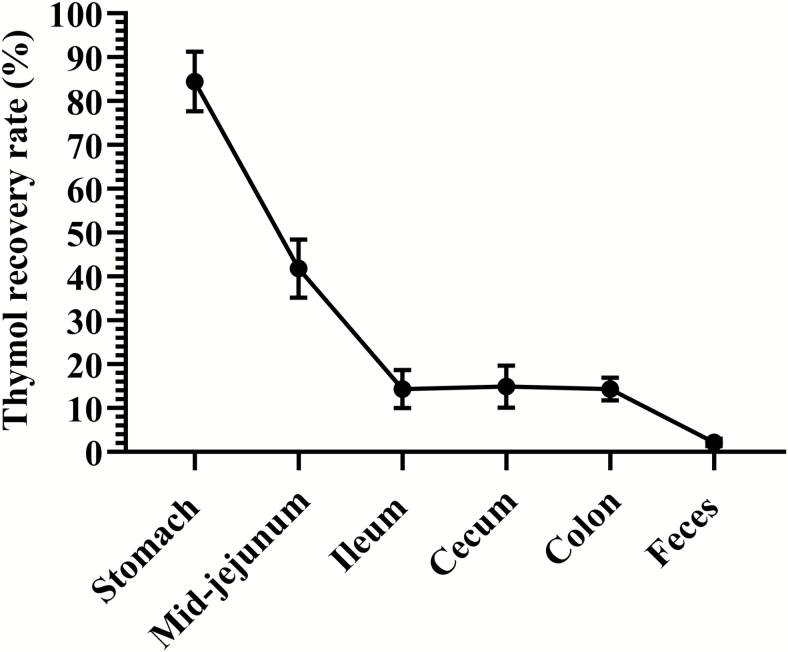
The recovery rate of thymol along the gut of weaned pigs fed a diet either nonsupplemented or supplemented with thymol microencapsulated in the lipid matrix microparticles. Thymol was not detectable along the gut of weaned pigs fed a diet not supplemented with thymol microencapsulated in the lipid matrix microparticles. Each value represents the mean ± SEM, *n* = 6.

In vitro release profile of thymol from the lipid matrix microparticles were investigated in SGF and SIF. As shown in Figure 4, 26.04% thymol was released in SGF, and the rest of the loaded thymol was progressively released in SIF until completion, which was achieved by around 24 h. The recovery rate of thymol was determined along the gut of weaned pigs fed a diet either nonsupplemented or supplemented with 6 g/kg thymol microencapsulated in the lipid matrix microparticles. During the whole experiment period, all pigs were healthy and consumed the feed at the normal quantity. The average final BW of all the pigs was 11.5 ± 0.99 kg and daily feed intake (days 6–9) was 0.45 ± 0.12 kg. There was no significant difference between the CF and EOF in the final BW and daily feed intake (*P* > 0.05). Thymol was not detectable along the gut of weaned pigs fed a diet nonsupplemented with thymol microencapsulated in the lipid matrix microparticles. As shown in Figure 5, 15.5% of thymol was released in the stomach, and 41.1% of thymol was delivered to the mid-jejunum section. The thymol was recovered in the ileum, cecum, and colon at 14.36%, 14.92%, and 14.35%, respectively. Only 2.21% of thymol was recovered in feces.

## DISCUSSION

Encapsulation, defined as a process in which microparticles or nanoparticles or droplets are encircled by a coating or embedded in a homogeneous or heterogeneous matrix, is a helpful method to improve the potency of feed additives (Gharsallaoui et al., 2007). The benefits of encapsulation are to 1) improve the storage stability of feed additives; 2) protect feed additives during feed processing, including mixing, conditioning, and pelleting; 3) mask unpleasant odor that can decrease feed intake; 4) improve the ease of handling of liquid feed additives (e.g., EO) by changing liquid to solid state; 5) slowly release bioactive compounds along the gut of animals; and 6) reduce the effective dosage of bioactive compounds that have high cost and environmental issues. A broad range of bioactive compounds, such as probiotics (Liu et al., 2016), EO (Omonijo et al., 2018a), zinc oxide (Xie et al., 2011), OA (Grilli et al., 2010), bacteriophages (Huff et al., 2013), and enzymes (Chandrasekar et al., 2017) have been encapsulated for improving animal health.

An ideal encapsulation should not only increase the stability of EO but also release them specifically in the target regions of the intestine (Chen et al., 2017). Wall or matrix materials are one of the most influential factors in controlling the release of bioactive compounds (Carneiro et al., 2013). Many wall or matrix materials, including polysaccharides (alginate xanthan gum), proteins (whey protein and gelatin), and lipids (milk fat and hydrogenated fat), have been used to encapsulate EO for effective delivery to the gut (Piva et al., 2007; Liu et al., 2016; Zhang et al., 2016; Chen et al., 2017). The benefits of encapsulated EO with hydrogenated fat are to facilitate slow release (Mehnert and Mäder, 2012) and to have high stability (Souto and Müller, 2010). Furthermore, solid lipid has been considered as the most cost-effective material for encapsulating EO. Solid lipid has also been used for encapsulating probiotics (Okuro et al., 2013), zinc oxide (Jang et al., 2014), vitamin A (Jenning et al., 2000), and OA (Piva et al., 2007). However, the stability of EO during feed processing and storage and the intestinal release of EO in animals are still not clear. Therefore, this study evaluated the stability of thymol in lipid matrix microparticles during a feed pelleting process and feed storage and determined the intestinal release of thymol using in vitro and in vivo approaches.

In modern farming, pig diets are commonly provided in a pellet form (Fahrenholz, 2012). The pelleting process is mainly composed of conditioning and pelleting. Conditioning refers to adding steam and heat to improve the binding property, while the purpose of pelleting is to agglomerate small particles into large particles (Falk, 1985). It has been proven that pelleting pigs’ diets enhance palatability, growth performance, nutrient, and energy digestibility and feed utilization efficiency compared to mash feeding (Vukmirović et al., 2017; Lahaye et al., 2008). However, the side effects of pelleting, including the possibility of breaking down of nutrients and feed additives, should be considered (Lewis et al., 2015; Kiarie and Mills, 2019). The most negative effects of pelleting are from wet steam, fat addition, and high energy input, which can decrease the stability of nutrients and feed additives (Broz et al., 1997). For example, Jongbloed and Kemme (1990) showed that when the pelleting temperature reached over 80 °C, the activity of exogenous phytase was decreased in the animal feed. In this study, the pelleting process did not change total thymol in the feed. The melting point of hydrogenated vegetable oil (matrix materials of the lipid matrix microparticles) is between 50 and 54 °C and the range of the measured temperature during the conditioning and pelleting process in this experiment reached between 61 and 74 °C. However, the pelleting process did not break down the lipid matrix microparticles and evaporate thymol in lipid matrix microparticles. There are several potential reasons: 1) the conditioning and pelleting time (less than 2 min for pelleting 50 kg) in this experiment was not long enough to break down the lipid matrix microparticles and to evaporate thymol; 2) feed ingredients possibly protected the lipid matrix microparticles during the conditioning part of the pelleting process in this experiment; and 3) after being melted during pelleting, lipids might still be with thymol together and then become solid particles again after pelleting. However, different pelleting conditions (e.g., higher temperature and longer time) may be able to break down the lipid matrix microparticles and evaporate EO. More studies are required to understand the effects of the pelleting process on the recovery rate of EO in lipid matrix microparticles with diverse pelleting conditions.

It was expected that there should be free thymol released from the lipid matrix microparticles but remained in the pelleted feed because pelleting aggregates the feed ingredients, which may inhibit the instant evaporation of thymol. The released thymol in the pelleted feed would be evaporated as when the pelleted feed was stored for 12 wk. However, because the amount of thymol in the pelleted feed did not change, it can be deduced that lipid matrix microparticles remained intact during the commercial pelleting process. In the swine industry, compound feed is stored for up to 3 mo before it is used. The free form of EO is vulnerable to oxidation by air and light (Moghaddam and Mehdizadeh, 2016). Furthermore, a study from Luo et al. (2005) showed that there are some mineral sources, including copper in the animal feed, which can accelerate the oxidation of bioactive compounds. In this experiment, EO encapsulated with hydrogenated vegetable oil maintained their stability during the storage and after mixing with other ingredients and pelleting. A potential reason is that hydrogenated vegetable oil, used as a matrix material in the experiment, may be resistant to oxidation and can maintain solid because its melting point is between 50 and 54 °C (Gupta, 2017). According to Mavromichalis and Baker (2000), harsh environmental conditions can be applied to feed in animal rooms where the temperature increases to more than 39 °C and during storage in silos and normal storage areas during the summer months (Alabadan and Oyewo, 2005), indicating the need for more storage stability studies in high-temperature environments.

In this study, lipid matrix microparticles could maintain their stability in SGF (pH 3) and released most of the EO in SIF. This is because lipids cannot be digested by pepsin and only digested by lipase with emulsification by bile salts in intestinal pH (e.g., pH 6–7) (Hussain et al., 2015). The 26.04% of released thymol in SGF may include the solubilized EO that existed on the surface of the microparticles and released EO from the physical pressure of shaking 2 h in SGF. While lipase in SIF may have played a critical role to break down the lipid matrix particles in SIF, bile salts also may have played an important function by emulsifying the lipid matrix microparticles, which generated new surfaces of the lipid matrix microparticles and facilitated the digestion of the lipid matrix microparticles (Schonewille et al., 2016). In agreement with the in vitro release study, Hamoudi et al. (2011) showed that it took approximately 24 h to release lipophilic drugs (progesterone) from the lipid beads made of α-cyclodextrin and soybean oil in SGF and SIF. It is important to note that there was a difference in the release profile when EO were encapsulated with different wall or matrix materials. A study by Zhang et al. (2016) showed that EO encapsulated with alginate-whey protein was released at approximately 20%–30% in the SGF incubation after 2 h and completely released at 6 h (SGF 2 h + SIF 4 h). Omonijo et al. (2018a) showed that approximately 50% of thymol encapsulated with starch and alginate were released within 2 h of incubation in SGF and 100% release was observed following incubation in SIF for an additional 2 h. These differences might be due to using different wall/matrix materials or differences in in vitro experimental conditions, such as enzyme concentrations and incubation temperature.

In the present study, 15.5% of thymol was released in the stomach and 41.85% of thymol was delivered to the mid-jejunum section and only 2.21% of thymol was recovered in the feces, which is considered a slow release. A slow release can be defined as releasing minimal amounts of bioactive compounds in the stomach and delivering high amounts of such compounds to the mid-jejunum section and releasing most of the bioactive compounds before they are excreted. Zhang et al. (2014) showed that approximately 38%, 19%, and 4% of the nonencapsulated form of carvacrol (e.g., EO) was recovered in the stomach, duodenum, and jejunum of weaned pigs, respectively, which indicated that significant amount of carvacrol disappeared in the stomach. Thus, as 84.5% of thymol was recovered in the stomach in the study, it can be inferred that only a minimal release occurred, thus, indicating a slow release.

A nutrient with digestibility of more than 90% is considered as very digestible for pigs and, thus, 2.21% of remained thymol in the feces indicates that almost all of the thymol disappeared in the gut of pigs (Jørgensen et al., 2000). In the in vivo study, 15.5% of the released thymol in the stomach may have included the solubilized thymol from the surface of the lipid matrix microparticles and released thymol from the physical pressure of the segmentation movement of the stomach. Also, some of the lipid matrix microparticles may have been digested by gastric lipase, which is secreted from gastric chief cells in the fundic mucosa and plays an important role in the digestion of lipid, especially in piglets. The possible reason for the difference between released thymol in SGF (SIF 0, 26.04%) and the stomach in pigs (15.5%) would be that SIF 0 represents finished incubation in the SGF 2 h, but a recovery rate of thymol in the stomach represents the released thymol during incubation in the stomach. Therefore, it would be more accurate to calculate the thymol recovery rate in the duodenum, but it was not feasible to collect duodenal digesta from piglets.

Most of the thymol from the lipid matrix microparticles was released in the jejunum, which can be estimated by subtracting the recovery rate of the ileum (14.36%) from the stomach (85.5%). Pancreatin enzymes, including lipase and bile salts, are secreted into the duodenum and most of the lipid sources are digested before they reach the ileum (Valette et al., 1992). However, the recovery rates of thymol in the ileum (14.36%), cecum (14.91%), and colon (14.35%) were similar. The potential explanation could be that 14.36% of thymol in the ileum existed as released form but thymol was not absorbed in the ileum, cecum, and colon and, after digesta were excreted as feces, most of the thymol was evaporated. There have been a few in vivo studies that have investigated the release profile of EO in pigs. In one of those studies, when EO microparticles encapsulated with alginate-whey protein were supplemented to pig, roughly 75%, 68%, 51%, 17%, 5%, and 5% were recovered in the stomach, duodenum, jejunum, ileum, cecum, and colon, respectively (Zhang et al., 2014). According to Piva et al. (2007), encapsulated OA and natural identical flavors with hydrogenated vegetable lipids showed a gradual decrease in the gastrointestinal tract (stomach, cranial jejunum, caudal jejunum, ileum, and cecum) of growing pigs compared with the nonencapsulated form of OA and natural identical flavors. As the lipid matrix microparticles were digested, some of the released thymol possibly showed beneficial properties, such as antimicrobial, antioxidative, and anti-inflammatory effects in the gastrointestinal tract of weaned pigs. Furthermore, some of the released thymol was most likely absorbed as secondary metabolites (thymol sulfate and thymol glucuronide) through the intestinal wall and transported by the blood to the liver (Pisarčíková et al., 2017). Therefore, the in vivo release experiment showed that the lipid matrix microparticles maintained their stability in the stomach and slowly released most of the thymol in the small intestine and delivered some thymol to the large intestine.

The current study shows that the lipid matrix microparticles can maintain stability during the pelleting process and storage. In vitro and in vivo release experiments demonstrated that the lipid matrix microparticles allowed for a slow release in simulated digestive fluids and along the gut of weaned pigs. The efficacy of lipid matrix microparticles in vivo has recently been validated by Xu et al. (2019) in weaned pigs challenged with enterotoxigenic *Escherichia coli* F4 (K88) by measuring growth performance and gut barrier function. However, more research is needed to investigate the effects of lipid matrix microparticles on nutrient absorption, immune responses, and microbiota in weaned pigs experimentally infected with *E. coli* F4 (K88).
